# Evaluation of DESS as a storage medium for microbial community analysis

**DOI:** 10.7717/peerj.6414

**Published:** 2019-02-05

**Authors:** Kevin M. Lee, Madison Adams, Jonathan L. Klassen

**Affiliations:** Department of Molecular and Cell Biology, University of Connecticut, Storrs, CT, USA

**Keywords:** DESS, Microbiome, Microbial ecology, Preservative, Fungus-growing ants

## Abstract

Microbial ecology research requires sampling strategies that accurately represent the microbial community under study. These communities must typically be transported from the collection location to the laboratory and then stored until they can be processed. However, there is a lack of consensus on how best to preserve microbial communities during transport and storage. Here, we evaluated dimethyl sulfoxide, ethylenediamine tetraacetic acid, saturated salt (DESS) solution as a broadly applicable preservative for microbial ecology experiments. We stored fungus gardens grown by the ant *Trachymyrmex septentrionalis* in DESS, 15% glycerol, and phosphate buffered saline (PBS) to test their impact on the fungus garden microbial community. Variation in microbial community structure due to differences in preservative type was minimal when compared to variation between ant colonies. Additionally, DESS preserved the structure of a defined mock community more faithfully than either 15% glycerol or PBS. DESS is inexpensive, easy to transport, and effective in preserving microbial community structure. We therefore conclude that DESS is a valuable preservative for use in microbial ecology research.

## Introduction

Microbial ecologists frequently use culture-independent methods to study the structure of microbial communities (Hugerth & Andersson, 2017). Such experiments face many design challenges that must be considered so that data they produce matches the structure of the original microbial community (Vandeputte et al., 2017; Pollock et al., 2018). Sample collection, handling, and storage are the first steps in any culture-independent study, and decisions made during this phase of a study can strongly affect downstream analyses (Hugerth & Andersson, 2017; Vandeputte et al., 2017). Ideally, DNA should be extracted from samples immediately following collection (Rochelle et al., 1994; Cardona et al., 2012). However, sampling typically occurs outside of the laboratory and samples can therefore experience prolonged and sometimes poorly controlled transportation and storage conditions that permit nucleic acid degradation and/or microbial growth (Amir et al., 2017). Either of these processes will alter the structure of the sampled microbial community such that obtained experimental data does not match the original structure of that community.

Cold storage (at −80, −20, or 4 °C, in order of preference) is the accepted gold standard for protecting samples from potentially adverse conditions that compromise microbial community structure (Rissanen et al., 2010; Williamson et al., 2011; Choo, Leong & Rogers, 2015; Hale et al., 2016; Kia et al., 2016; Song et al., 2016, but see also Bahl, Bergström & Licht, 2012). One disadvantage of cold storage is that cold chains are fragile, especially when collection sites are remote (Vandeputte et al., 2017). Electrically-powered cooling requires substantial and robust physical infrastructure. Alternatively, refrigerants such as ice, dry ice, and liquid nitrogen can be difficult to obtain, must be refreshed routinely, and (in the case of dry ice and liquid nitrogen) are regulated or prohibited on many modes of transportation. These issues limit the deployment and robustness of cold storage for preserving microbial community samples, despite its effectiveness.

Many studies have used preservative media as an alternative solution to overcome the logistical difficulties of cold transport and storage. These include proprietary media such as DNAgard, RNAlater, OMNIgene.GUT, and LifeGuard, and less-expensive non-proprietary media such as Tris-EDTA, ethanol, and phenol-chloroform (Rissanen et al., 2010; Gaither et al., 2011; Gray, Pratte & Kellogg, 2013; Tatangelo et al., 2014; Choo, Leong & Rogers, 2015). As with cold storage, these buffers attempt to preserve the structure of the microbial community being sequenced. Many studies have compared the effectiveness of preservative media for a variety of sample types (Rissanen et al., 2010; Vlčková et al., 2012; Gray, Pratte & Kellogg, 2013; Tatangelo et al., 2014; Choo, Leong & Rogers, 2015; Anderson et al., 2016; Blekhman et al., 2016; Amir et al., 2017; Vogtmann et al., 2017), and typically conclude that most preservatives alter microbial community structure to a greater or lesser degree. Some preservatives such as ethanol and phenol-chloroform are also hazardous and therefore face travel restrictions, limiting their use for field collections. There is therefore an ongoing need to identify storage media that are effective, inexpensive, and well-suited to field collections.

Dimethyl sulfoxide (DMSO)-ethylenediamine tetraacetic acid (EDTA)-saturated salt solution (DESS, also known as SSD) is a non-proprietary, non-hazardous storage medium that shows strong potential for use as a preservative in microbial ecology studies. In this medium, DMSO permeates cells and facilitates the rapid entry of EDTA that suppresses nuclease activity by chelating divalent cations. Sodium chloride salt further suppresses enzymatic activity and contributes sodium ions that stabilize the negatively charged phosphate backbone of DNA (Seutin, White & Boag, 1991). DESS was first used to preserve avian blood samples by Seutin, White & Boag (1991) and has since been used to preserve the morphology and DNA of nematodes (Yoder et al., 2006), corals, and small marine invertebrates (Gaither et al., 2011). DESS did not strongly alter microbial community structure when used to preserve mock microbial communities created from environmental isolates (Gray, Pratte & Kellogg, 2013) or soil and water samples, with and without cold storage (Tatangelo et al., 2014). Other DMSO-based preservatives have similar properties (Kerckhof et al., 2014). DESS therefore shows promise as a broadly applicable preservative that can overcome the fragility or lack of cold chains during sample transport between collection sites and the laboratory.

Here, we evaluated DESS as a preservative using the microbial communities found in fungus gardens grown by the ant *Trachymyrmex septentrionalis* as a model system studied by our research group. We typically collect these samples in hot and humid locations that are far from the lab, meaning that our cold chain is susceptible to failure. We further validated our field-based observations using a mock microbial community with a defined structure. Our results suggest that DESS is an excellent preservative of microbial community structure that is useful for field collections where cold transport and storage are challenging.

## Materials and Methods

### Sample collection

*Trachymyrmex septentrionalis* colonies were collected in New Jersey, Florida, Georgia, and North Carolina during 2014 and 2015. Permits for collecting samples were obtained from the corresponding state department: State of New Jersey Department of Environmental Protection Division of Parks and Forestry State Park Service Unnumbered Letter of Authorization; North Carolina Division of Parks and Recreation Scientific Research and Collecting Permit 2015_0030; Florida Department of Agriculture and Consumer Services unnumbered Letter of Authorization; Georgia Department of Natural Resources State Parks & Historic Sites Scientific Research and Collection Permit 032015; Georgia Department of Natural Resources Wildlife Resources Division unnumbered Letter of Authorization. *T. septentrionalis* colonies were identified by their distinctive “half-moon” mound shape and the presence of *T. septentrionalis* worker ants. An initial ∼25 cm deep trench was dug beside each colony entrance and then expanded until the fungus garden chamber was gently breached. After expanding this opening, the fungus garden was removed using a flame-sterilized spoon. Fungus gardens were naturally homogenized during collection by crumbling due to their fragility. Approximately 200 mg of each fungus garden was subsampled into DESS (20% DMSO (v/v), 250 mM EDTA, saturated with sodium chloride), 15% (v/v) glycerol, or PBS (137 mM NaCl, 2.7 mM KCl, 10 mM Na_2_HPO_4_, 2 mM KH_2_PO_4_, pH = 7.4), frozen immediately on dry ice, and then transferred to −80 °C storage upon return to the laboratory. Glycerol was chosen for comparison based on its potential use as a cryoprotectant that would allow cells to be cultured upon return to the lab. PBS served as a baseline that was not expected to facilitate sample preservation beyond maintaining osmotic balance. The *Atta texana* colony used to generate data for Fig. S1 was collected from Louisiana in 2016 under Louisiana Department of Wildlife and Fisheries permit WL-Research-2016-10. This colony was already being maintained in the laboratory at the time of sampling. A small (5 × 5 × 5 cm) piece of the fungus garden was homogenized in a sterile petri dish and sampled in the same manner using DESS, PBS, and 100% ethanol. These samples were immediately placed in the −80 °C freezer for 10 days prior to processing.

### Sample processing

#### Fungus gardens

Samples were warmed to −20 °C overnight and then thawed at 4 °C just prior to processing (2–5 hours). Replicate subsamples were pooled to obtain ∼1 g wet mass of fungus garden (equivalent to 250–500 mg dry mass). We adapted the protocol of Apajalahti et al. (1998) to enrich for bacterial cells prior to DNA extraction, using medium speed vortexing to homogenize samples instead of rotational shaking and a final centrifugation at 15,000×*g* for 15 min instead of 30,000×*g* to pellet bacterial cells. DNA was extracted from the resulting cell pellets using a CTAB/bead beating protocol modified from Cafaro & Currie (2005) by using three 2-min cycles of bead beating (Biospec Minibeadbeater, Bartlesville, OK, USA) separated by 2.5 min cooling on ice for cell lysis and 24:1 chloroform:isoamyl alcohol for DNA extraction. DNA extracts were resuspended in nuclease-free water and quantified spectrophotometrically using a BioSpec EON plate reader with a Take3Trio plate. Negative DNA extraction controls that contained water instead of sample were included alongside each extraction batch. DNA extracts with A_260/280_ or A_260/230_ ratios ≤1.5 were purified using Agencourt XP magnetic beads (Beckman Coulter, Brea, CA, USA) following the manufacturer’s protocol.

#### Mock community

A ZymoBIOMICS Microbial Community Standard (Zymo Research Corporation, Irvine, CA, USA) composed of five Firmicutes (Gram positive) and three Proteobacteria (Gram negative) was used to precisely test DESS’s ability to preserve microbiome DNA. Although not as diverse as *T. septentrionalis* fungus gardens, the major constituents of the mock community belong to the predominant phyla that we observed in the *T. septentrionalis* fungus gardens (see Results). One vial of this mock community was divided into 18 equal aliquots and each aliquot was centrifuged at 5,000×*g* for 5 min to pellet the cells. The supernatant containing the proprietary Zymo preservative was removed and the cells were resuspended in one ml of DESS, PBS, or 15% glycerol by vortexing. DNA was extracted from two samples resuspended in each preservative as described above either immediately (before freezing; *t*_0_) or after one (*t*_1_) or two (*t*_2_) months of storage at −80 °C. These DNA extracts were quality checked as described above but none required magnetic bead cleaning. Negative controls were not used for this experiment because of the known composition of the mock communities.

#### PCR screening

All quality-checked DNA extracts were screened by PCR amplification of the V4 region of the 16S rRNA gene before sequencing on an Illumina MiSeq. Each 25 μl reaction used 1X GoTaq reaction buffer (Promega, Madison, WI, USA), 0.3 μM each of primers 515F and 806R (Caporaso et al., 2011; Invitrogen, Carlsbad, CA, USA), 1.25 units of GoTaq DNA polymerase (Promega, Madison, WI, USA), 300 ng/μl bovine serum albumin (New England BioLabs, Ipswich, MA, USA), 50 ng of template DNA, and nuclease-free water (Thomas Scientific, Swedesboro, NJ, USA). PCR reactions were run on a T-100 Thermal Cycler (BioRad, Hercules, CA, USA) for 3 min at 95 °C, 30 cycles of: 30 s at 95 °C, 30 s at 50 °C, and 60 s at 72 °C, followed by a single 5 min cycle at 72 °C. Bands were visualized using agarose gel electrophoresis. PCR reactions lacking the expected 350 bp product were re-cleaned using magnetic beads as described above and screened by PCR a second time.

### Community amplicon sequencing

Quality-checked, PCR-screened DNA extracts were submitted to the University of Connecticut Microbial Analysis, Resources, and Services (MARS) facility for sequencing. Submitted DNA samples were quantified fluorometrically using a PicoGreen (Invitrogen, Carlsbad, CA, USA) assay in 384-well plates read on a Synergy plate reader (BioTek, Winooski, VT, USA). After quantification, 30 ng of sample DNA was added to 1× Phusion High Fidelity master mix (New England Biolabs, Ipswich, MA, USA) containing 1 μM indexed sequencing primers with Illumina adapters (Kozich et al., 2013; Invitrogen, Carlsbad, CA, USA), four nM non-Illumina primers (515F/806R; Caporaso et al., 2011; Invitrogen, Carlsbad, CA, USA) to a final volume of 50 μl. These reactions were split into three equal aliquots and PCR amplified using settings: 94 °C for 3 min initial denaturation, 30 cycles of 94 °C for 45 s denaturation, 50 °C for 1-min annealing, 72 °C for 1.5 min extension, and 72 °C for 10 min final extension. These PCR reactions were re-pooled and quantified using a QIAxcel instrument (Qiagen, Venlo, Netherlands). Sample libraries with a PCR product concentration >0.5 ng/μl and peak(s) at 400 bp (±15%) were pooled by adding equal masses of PCR product from each sample. These pooled libraries were cleaned using Mag-Bind RXNPure Plus beads (OMEGA bio-tek, Norcross, GA, USA), resuspended in 25 μl molecular biology grade water, quantified using a Qubit assay (Invitrogen, Carlsbad, CA, USA), and adjusted to 4 nM (1.5 ng/μl when using MARS adaptors). Amplicon libraries were diluted to 6 pM in Illumina HT1 buffer with 30% PhiX phage DNA added and sequenced using a V2 (2 × 250) cartridge on an Illumina MiSeq instrument.

### Bioinformatic analysis

16S rRNA gene sequences were processed in R v3.4.3 (R Development Core Team, 2017) using the DADA2 v1.7.0 pipeline (Callahan et al., 2016) following the guidelines at https://benjjneb.github.io/dada2/tutorial.html (accessed 7/2/2018). Processed sequences and accompanying metadata were imported into phyloseq v1.22.3 (McMurdie & Holmes, 2013) and screened for potential contaminants using decontam v0.20.0 (Davis et al., 2018) using the decontam “prevalence protocol” with the P* threshold set to 0.5. Amplicon sequence variants (ASVs) that were not classified as bacteria were removed, as were bacterial sequences that were not classified to at least the phylum level. Decontam identified 36 fungus garden ASVs as contaminants (ranging from 0% to 10.2% of the sequences in each sample, mean 1.4%), which were removed from this dataset. After excluding four samples with low read counts, the final fungus garden dataset contained 26 samples from 10 *T. septentrionalis* colonies (minimum: 11,379 reads; mean: 46,125 reads). Instead of using decontam, seven ASVs (ranging from 0.4% to 3.4% of the sequences in each sample, mean 1.6%) were manually detected and removed from the mock community dataset because they did not match known members of that community. All 16 mock community samples were included in the final mock community dataset (minimum: 24,891 reads; mean: 36,445 reads). The raw sequence data have been deposited in the National Center for Biotechnology Information database under BioProject ID PRJNA479679.

The resulting phyloseq-compatible R data object was subsampled to the lowest read count among all libraries and ASV counts were converted to relative abundances. Weighted UniFrac distances were calculated, ordinated, and visualized using the distance, ordinate, and plot_ordination functions in phyloseq, respectively. Phyloseq was also used to calculate Shannon’s diversity, Chao1 diversity, and the observed diversity metrics using the estimate_richness function. Permutational analysis of variance (PERMANOVA) tests were done using Adonis in the vegan R package v2.4–5 (Oksanen et al., 2017). The aov, kruskal.test, and TukeyHSD commands implemented in the base R package were used to perform analysis of variance (ANOVA), Kruskal–Wallace and Tukey’s honest significant difference testing, respectively. The code and metadata files to reproduce these analyses are available in File S1.

## Results

### Ant fungus gardens

In earlier work sequencing 16S rRNA genes in *T. septentrionalis* fungus gardens, Ishak et al. (2011) reported difficulty in generating sequencing reads from ethanol-preserved fungus garden samples. Similarly, our extractions of fungus gardens preserved in ethanol consistently yielded low-quality DNA (see Fig. S1, DNA extractions from *A. texana* fungus gardens). DNA extracts from ethanol-preserved fungus garden samples had a green-brown discoloration when compared to either PBS- or DESS-preserved samples, which were both visibly clear (Fig. S1A). Ethanol-preserved samples yielded more DNA than DESS- or PBS-preserved samples, but this DNA was highly fragmented (Fig. S1B). PCR amplification of the 16S rRNA gene V4 region from these ethanol-preserved samples was unsuccessful (Fig. S1C). Our results agreed with previous work (Rissanen et al., 2010; Gaither et al., 2011) that indicated poor preservation of DNA when stored in ethanol. We therefore discontinued the use of ethanol as a preservative for our microbiome characterization studies and instead focused on DESS as a safe and inexpensive alternative to ethanol for preservation of microbial community DNA during our field work.

**Figure 1:**
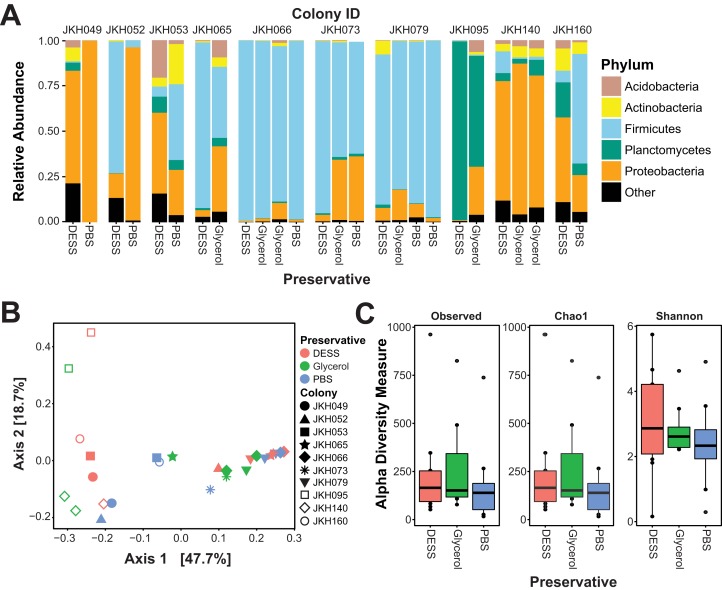
Preservative type does not alter the community structure of *T. septentrionalis* fungus gardens. (A) Relative abundances of phyla in *T.* septentrionalis fungus gardens, grouped by colony. Phyla with relative abundances ≤15% in all samples are grouped into a single “Other” category. (B) PCoA of Weighted UniFrac distances between *T. septentrionalis* fungus garden bacterial communities. Colors indicate preservative types, and shapes indicate samples from different colonies. (C) Alpha-diversity (Shannon’s, Observed, and Chao1) in *T. septentrionalis* fungus gardens. Center bars in the box plot indicate the median, top and bottom of boxes indicate the 25th and 75th quartile, respectively, and whiskers indicate ±1.5 times the standard error. Single points represent outliers.

To evaluate DESS as a preservative of microbial community DNA, we compared *T. septentrionalis* fungus garden samples stored in DESS to parallel samples stored in either PBS as a reference control or glycerol as a cryoprotectant that was not expected to prevent DNA degradation. Compared to between-colony differences, the type of preservative in which samples were stored only minimally correlated with variation in microbial community structure. Principal coordinate analysis (PCoA) of both Weighted and Unweighted UniFrac distances showed that samples from the same colony group together. In contrast, samples stored in different media are distributed throughout each PCoA plot (Fig. 1B—Weighted UniFrac, Fig. S2—Unweighted UniFrac). Colony of origin accounted for the largest amount of the between-sample variation in β-diversity (PERMANOVA Weighted UniFrac, *F* = 52.496, *R*^2^ = 0.799, *p* = 0.001, Unweighted UniFrac *F* = 2.2136, *R*^2^ = 0.555, *p* = 0.001) with storage medium accounting for a very small percentage of this variation in the Weighted but not the Unweighted Unifrac analysis (PERMANOVA Weighted UniFrac, *F* = 11.78, *R*^2^ = 0.039, *p* = 0.006, Unweighted UniFrac *F* = 0.84939, *R*^2^ = 0.069, *p* = 0.764) that was linked to colony of origin (colony-by-storage medium interaction: PERMANOVA Weighted UniFrac, *F* = 8.37, *R*^2^ = 0.156, *p* = 0.010, Unweighted UniFrac *F* = 1.0959, *R*^2^ = 0.311, *p* = 0.317). Weighted UniFrac distances between samples from the same colony that were stored in different preservatives did not significantly differ from each other (Kruskal–Wallace, D*f* = 2, χ^2^ = 4.779, *p* = 0.090), implying that one preservative type did not alter microbial community structure more than the others. Like β-diversity, the α-diversity of samples collected from the same colony did not vary between preservative types (Fig. 1C; Kruskal–Wallace, Shannon: D*f* = 2, χ^2^ = 0.934, *p* = 0.627; Chao1 and Observed: D*f* = 2, χ^2^ = 1.127, *p* = 0.569). Collectively, these data indicate that preservative type had at most a minor effect on the observed structure of *T. septentrionalis* fungus garden microbial communities.

### Mock community samples

**Figure 2:**
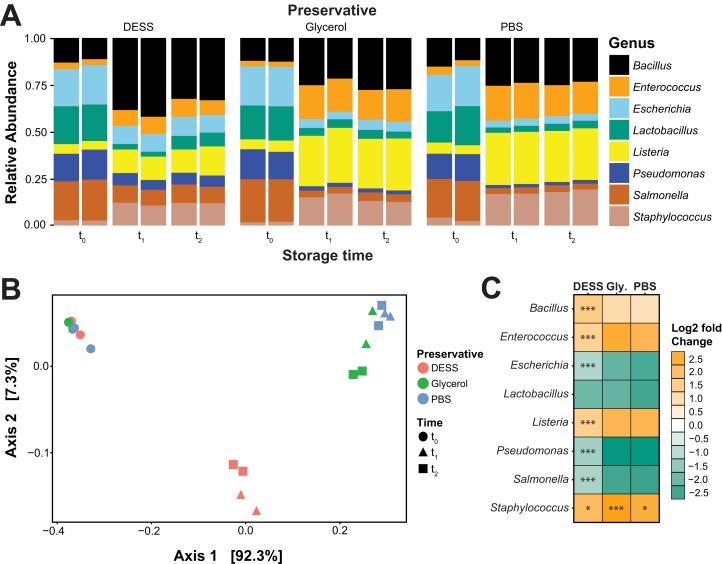
DESS preserves microbial mock community structure better than PBS or glycerol. (A) Relative abundances of genera in mock community samples. (B) PCoA of Weighted UniFrac distances between mock communities stored in DESS, PBS, or glycerol, indicated by different colors, for 0, 1, or 2 months, indicated by different shapes. (C) Heatmap showing log2 fold changes in relative abundance of genera in the mock community compared to *t*_0_ samples. *Indicate where these changes were significantly different between preservatives (Bonferroni corrected *p*-values: *p < 0.01, ***p < 0.001).

Our experiments with *T. septentrionalis* fungus gardens suggested that preservative type may have a small effect on community structure. However, the lack of a reference standard prevented us from determining which samples best represented the true structure of *T. septentrionalis* fungus garden microbial communities and which represented artificial community structures that were biased by the effects of storage. We therefore used a mock community of known composition to isolate the effect of preservative on microbial community structure. Storage at −80 °C for 1 or 2 months changed the relative abundances of all taxa in all preservatives when compared to the corresponding unfrozen (*t*_0_) samples (Fig. 2A). The PCoA of the Weighted UniFrac distances showed three distinct clusters, containing either: (1) all *t*_0_ samples; (2) *t*_1_ and *t*_2_ DESS samples; or (3) *t*_1_ and *t*_2_ glycerol and PBS samples (Fig. 2B). As with fungus garden samples, variation in mock community structure correlated minimally with preservative type (PERMANOVA, *F*_(2,17)_ = 163.8, *R*^2^ = 0.133, *p* = 0.001). Instead, variation in mock community structure was highly correlated to storage time (PERMANOVA, *F*_(2,17)_ = 977.5, *R*^2^ = 0.794, *p* = 0.010). The mean Weighted UniFrac distances between *t*_1_ and *t*_2_ samples frozen in the same preservative were very small (DESS = 0.054, glycerol = 0.055, PBS = 0.036) and not significantly different from each other (ANOVA, *F*_(2,18)_ = 1.959, *p* = 0.175). These small distances imply that “time” actually represents changes induced by freezing and/or any amount of frozen storage.

Mock community samples frozen in DESS changed less during storage at −80 °C compared to the samples that were stored in either PBS or glycerol (Fig. 2C). *Bacillus*, *Enterococcus*, *Listeria*, and *Staphylococcus* were all overrepresented in mock communities stored in all preservatives relative to the *t*_0_ baseline samples. Similarly, *Escherichia*, *Lactobacillus*, *Pseudomonas*, and *Salmonella* were all underrepresented in mock communities stored in all preservatives relative to the *t*_0_ baseline samples. The relative abundance of six out of eight taxa differed from the *t*_0_ baseline samples less when frozen in DESS compared to glycerol and PBS (Fig. 2C). The exceptions were *Lactobacillus,* whose relative abundance changed equally in all preservatives, and *Bacillus*, whose relative abundance changed more in DESS compared to the glycerol and PBS. Changes in taxon relative abundance compared to the *t*_0_ baseline did not differ between taxa stored in glycerol or PBS (Tukey’s Honest Significance with Bonferroni correction, *p* > 0.050), except for *Staphylococcus* (Tukey’s Honest Significance with Bonferroni correction, *p* = 0.016). Collectively, these data show that although freezing changes the structure of all mock community samples, communities stored in DESS were more similar to that of unfrozen *t*_0_ baseline samples compared to samples stored in glycerol or PBS.

## Discussion

*Trachymyrmex septentrionalis* fungus garden microbial communities were more diverse than what has been reported for fungus gardens from several related ant species. Fungus-growing ants from the genera *Atta* and *Acromyrmex* raise fungus gardens that contain low microbial diversity and that are dominated by Gammaproteobacteria, as are fungus gardens grown by *T. zeteki* (Suen et al., 2010; Aylward et al., 2012, 2014). In contrast, our results suggest that *T. septentrionalis* fungus garden microbial communities are highly variable (Fig. 1A). Of the 10 sampled fungus gardens, four were dominated by Firmicutes, two were dominated by Proteobacteria, and one was dominated by Planctomycetes. The remaining three colonies had high amounts of both Firmicutes and Proteobacteria (Fig. 1A). Acidobacteria, Actinobacteria, Verrucomicrobia, and (except for colony JKH095) Planctomycetes had low abundances in most fungus gardens. Our results agree with the limited data available for other *T. septentrionalis* fungus gardens (Ishak et al., 2011) and some other ant genera (Kellner et al., 2015). These differences between *T. septentrionalis* and leaf-cutting ant fungus gardens could be due to species-specific differences in habitat and foraging behavior (De Fine Licht & Boomsma, 2010) that might lead to different microbes entering fungus gardens via different substrates. Although our limited dataset lacks sufficient statistical power to robustly identify ecological factors that structure *T. septentrionalis* fungus garden microbial communities, these aspects remain active and ongoing areas of research in our lab.

Preservative type had only a small impact on *T. septentrionalis* fungus garden microbial community structure. The ∼4% of variation in fungus garden microbial community structure attributed to preservative by PERMANOVA was dwarfed by the >75% of variation attributed to the colony of origin (Fig. 1B). Preservative type also did not correlate with changes in the alpha-diversity of *T. septentrionalis* fungus garden microbial communities (Fig. 1C). This minimal effect of preservative parallels other studies where between-host differences in microbial community structure greatly exceeded differences caused by variation in storage strategy (Bai et al., 2012; Hill et al., 2016; Song et al., 2016).

Because our *T. septentrionalis* fungus gardens were all frozen in the field, we lacked a reference community against which to compare the slightly different microbial community structures of the fungus gardens stored in DESS compared to those stored in glycerol or PBS. We therefore used a mock community to more precisely assess changes in microbial community structure caused by each preservative. In these experiments, all mock communities changed relative to *t*_0_ samples, likely indicating either a consistently negative effect of storage for 1–2 months at −80 °C (Bahl, Bergström & Licht, 2012; Kia et al., 2016) and/or the consistently negative effect of the additional freeze/thaw cycle undergone by the *t*_1_ and *t*_2_ samples but not the *t*_0_ samples (Cardona et al., 2012; Gorzelak et al., 2015). Future research into DESS should focus on how microbial community structure changes under fluctuating storage conditions, as might occur in a broken cold chain. Four of the five Firmicutes strains in the mock community were overrepresented after storage at −80 °C. Such increases in Firmicutes following cold storage has been reported previously (Bahl, Bergström & Licht, 2012; Anderson et al., 2016; Hill et al., 2016; Song et al., 2016). However, these changes were less severe for samples stored in DESS compared to those stored in glycerol or PBS, complementing previous studies that showed DESS to be an effective storage medium using different sample types and storage temperatures (Gray, Pratte & Kellogg, 2013; Tatangelo et al., 2014).

## Conclusions

In summary, our results suggest that DESS is an excellent storage medium for microbial ecology samples. Storage in DESS did not obscure the ecological differences between the microbial communities of fungus gardens collected from different ant colonies. DESS also preserved the community structure of a mock community more faithfully than either glycerol or PBS. DESS is inexpensive and non-hazardous, making it easy to transport. Together, these attributes suggest that DESS is a versatile and economical preservative that is suitable for the transport and storage of microbial communities.

## Supplemental Information

10.7717/peerj.6414/supp-1 Supplemental Information 1 Code and metadata for this study.

10.7717/peerj.6414/supp-2 Supplemental Information 2 Ethanol produces inconsistent results when used as a preservative for fungus garden samples. (A) DNA extracts from Ethanol (1–3), DESS (4–6) and PBS (7–9). Note the visible discoloration of the DNA extracts from ethanol preserved samples (B) DNA smears from Ethanol (lanes 3–5), DESS (lanes 7–9), and PBS (lanes 11–13). 5μl volumes of each sample loaded, 1% Agarose (Fisher) gel run at 40 volts for 150 minutes, 1kb DNA ladder (lane 1, Promega). (C) PCR amplification of the V4 region of the 16S rRNA gene, 1% Agarose (Fisher) gel run at 40 volts for 150 minutes, 100bp DNA ladder (lane 1, Promega) lane 1: 1kb DNA ladder (Promega), lane 2: Positive control (50ng *Escherichia coli* genomic DNA), lane 3: Negative PCR control, lanes 4–6: DESS samples, lanes 8–10: PBS samples, lanes 11–13: Ethanol samples.

10.7717/peerj.6414/supp-3 Supplemental Information 3 Preservative type does not alter the community structure of *T. septentrionalis* fungus gardens. PCoA of Unweighted UniFrac distances between *T. septentrionalis* fungus garden bacterial communities. Colors indicate preservative types, and shapes indicate samples from different colonies.

## Additional Information and Declarations

### Field Study Permissions

The following information was supplied relating to field study approvals (i.e., approving body and any reference numbers): Permits for collecting samples were obtained from the corresponding state department: State of New Jersey Department of Environmental Protection Division of Parks and Forestry State Park Service Unnumbered Letter of Authorization; North Carolina Division of Parks and Recreation Scientific Research and Collecting Permit 2015_0030; Florida Department of Agriculture and Consumer Services unnumbered Letter of Authorization; Georgia Department of Natural Resources State Parks & Historic Sites Scientific Research and Collection Permit 032015; Georgia Department of Natural Resources Wildlife Resources Division unnumbered Letter of Authorization; Louisiana Department of Wildlife and Fisheries permit WL-Research-2016-10.

### DNA Deposition

The following information was supplied regarding the deposition of DNA sequences: The raw sequence data are available at NCBI BioProject under BioProject ID PRJNA479679.

### Data Availability

The following information was supplied regarding data availability: The raw data (code and metadata files) are available in the Supplemental Files.
