# Supplementary figures and images for: Evaluation of DESS as a storage medium for microbial community analysis
Source: PeerJ. 2019 Feb 5;7:e6414. doi: 10.7717/peerj.6414 (PMC6368006; doi:10.7717/peerj.6414)

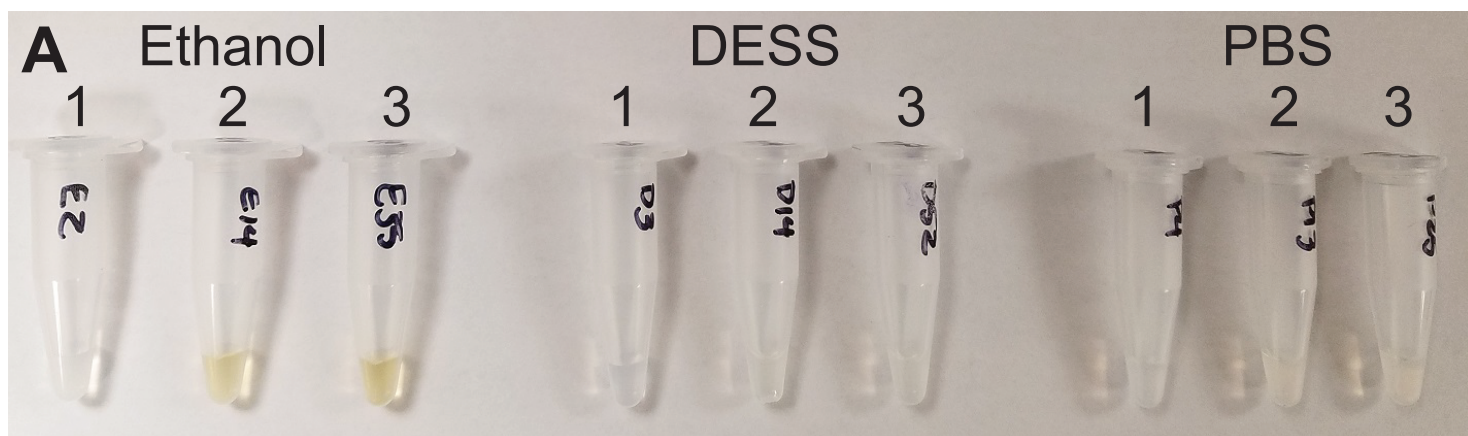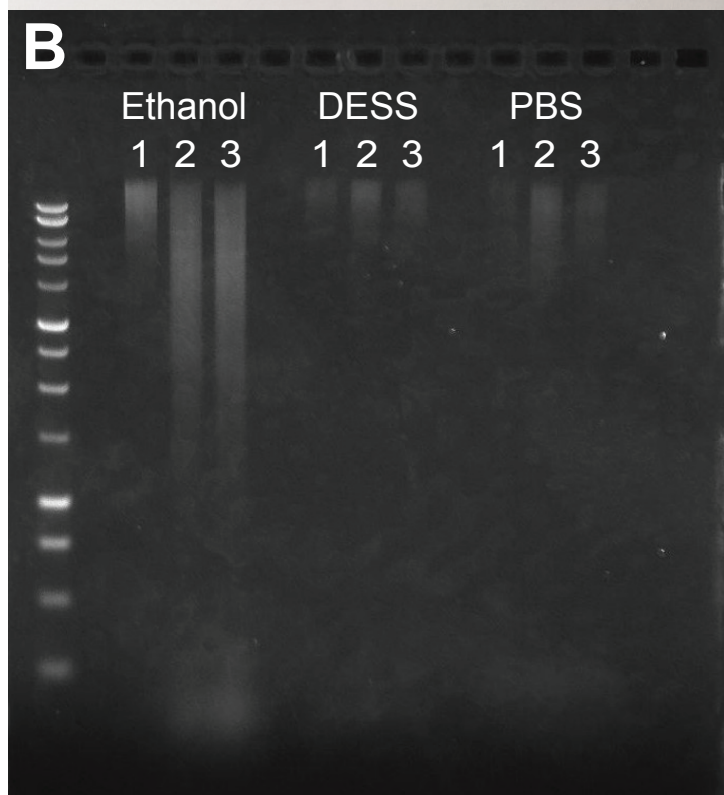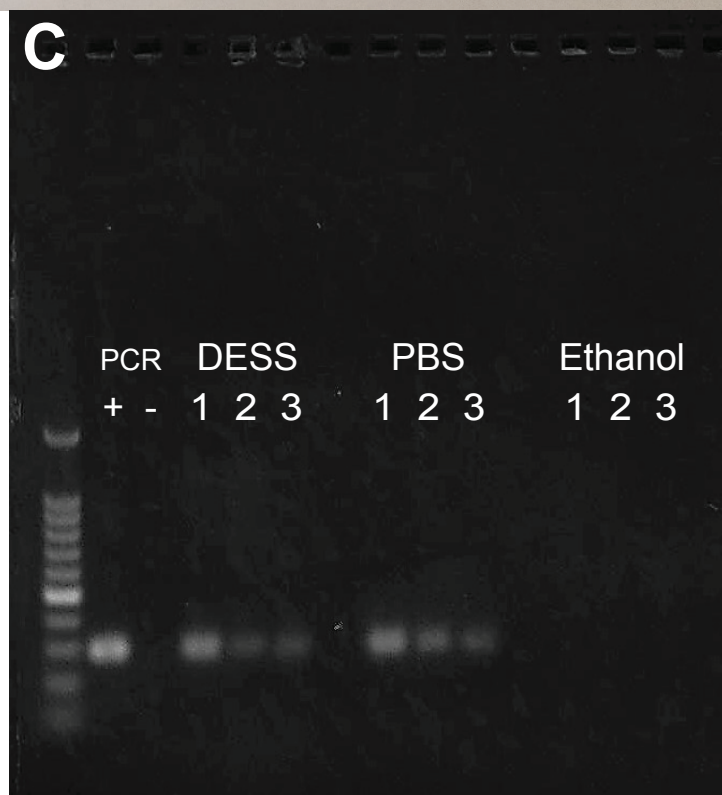

Supplement: Supplemental Information 2 — (A) DNA extracts from Ethanol (1–3), DESS (4–6) and PBS (7–9). Note the visible discoloration of the DNA extracts from ethanol preserved samples (B) DNA smears from Ethanol (lanes 3–5), DESS (lanes 7–9), and PBS (lanes 11–13). 5μl volumes of each sample loaded, 1% Agarose (Fisher) gel run at 40 volts for 150 minutes, 1kb DNA ladder (lane 1, Promega). (C) PCR amplification of the V4 region of the 16S rRNA gene, 1% Agarose (Fisher) gel run at 40 volts for 150 minutes, 100bp DNA ladder (lane 1, Promega) lane 1: 1kb DNA ladder (Promega), lane 2: Positive control (50ng Escherichia coli genomic DNA), lane 3: Negative PCR control, lanes 4–6: DESS samples, lanes 8–10: PBS samples, lanes 11–13: Ethanol samples. [file peerj-07-6414-s002.pdf]

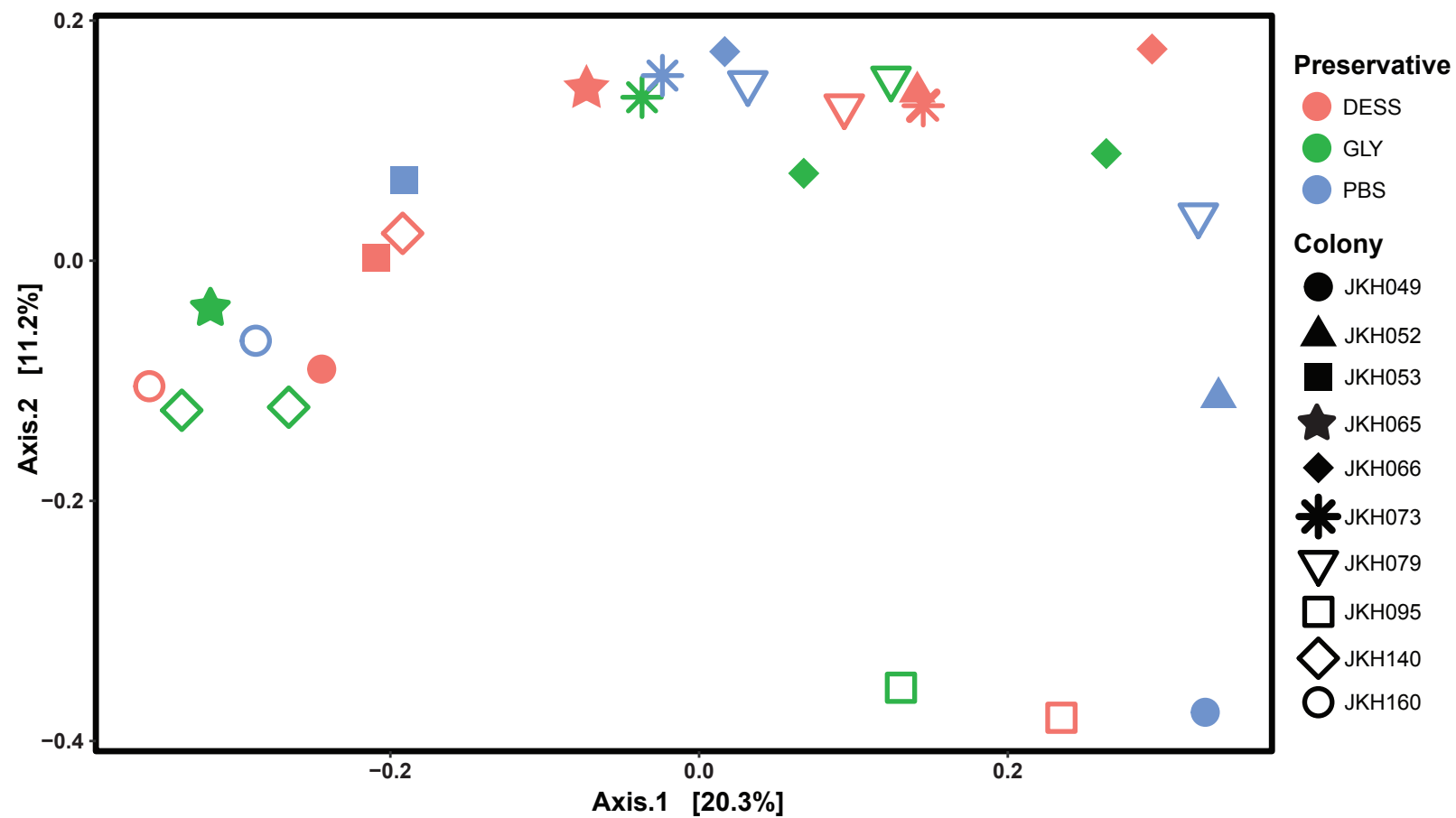

Supplement: Supplemental Information 3 — PCoA of Unweighted UniFrac distances between T. septentrionalis fungus garden bacterial communities. Colors indicate preservative types, and shapes indicate samples from different colonies. [file peerj-07-6414-s003.pdf]
